# Optimal Siting and Sizing of Multiple DG Units for the Enhancement of Voltage Profile and Loss Minimization in Transmission Systems Using Nature Inspired Algorithms

**DOI:** 10.1155/2016/1086579

**Published:** 2016-02-08

**Authors:** Ambika Ramamoorthy, Rajeswari Ramachandran

**Affiliations:** ^1^Department of Electrical Engineering, Anna University, Chennai, Tamil Nadu 600 025, India; ^2^Department of Electrical Engineering, Government College of Technology, Coimbatore, Tamil Nadu 641 041, India

## Abstract

Power grid becomes smarter nowadays along with technological development. The benefits of smart grid can be enhanced through the integration of renewable energy sources. In this paper, several studies have been made to reconfigure a conventional network into a smart grid. Amongst all the renewable sources, solar power takes the prominent position due to its availability in abundance. Proposed methodology presented in this paper is aimed at minimizing network power losses and at improving the voltage stability within the frame work of system operation and security constraints in a transmission system. Locations and capacities of DGs have a significant impact on the system losses in a transmission system. In this paper, combined nature inspired algorithms are presented for optimal location and sizing of DGs. This paper proposes a two-step optimization technique in order to integrate DG. In a first step, the best size of DG is determined through PSO metaheuristics and the results obtained through PSO is tested for reverse power flow by negative load approach to find possible bus locations. Then, optimal location is found by Loss Sensitivity Factor (LSF) and weak (WK) bus methods and the results are compared. In a second step, optimal sizing of DGs is determined by PSO, GSA, and hybrid PSOGSA algorithms. Apart from optimal sizing and siting of DGs, different scenarios with number of DGs (3, 4, and 5) and *PQ* capacities of DGs (*P* alone, *Q* alone, and  *P* and *Q* both) are also analyzed and the results are analyzed in this paper. A detailed performance analysis is carried out on IEEE 30-bus system to demonstrate the effectiveness of the proposed methodology.

## 1. Introduction

Today, the power grid is transforming and evolving into a faster-acting, potentially more controllable grid than in the past. This so-called smart grid will incorporate new digital and intelligent devices to replace the existing power network [1]. This grants an opportunity for new innovations and modernizations.

The massive penetration of distributed generation into electric grid is one of the salient features of smart grid. But the integration of DGs perturbs the power flow and voltage conditions of the network. So, voltage regulation is one of the major issues to be addressed [2].

The 16% of global final energy consumption comes from renewable sources during 2012, with 10% coming from traditional biomass, 3.4% coming from hydroelectricity, and the remaining 2.6% coming from new renewable sources like wind, solar power, and so forth [3].

Solar power takes the prominent position among all other sources due to its continuous availability and cost effectiveness. Solar energy is available in abundance [4]. But there are several challenges in adding renewable energy sources into the conventional grid [5]. The size and location of DGs are the crucial factors in the application of DG for loss minimization [6].

One of the key requirements for reliable electric power system operation is the balancing of reactive power supply and demand to maintain adequate system voltages. Lack of sufficient reactive power supplies can result in voltage instability. The peripheral method of balancing the reactive power and voltage of the system is to add capacitors, tap changing transformers, and FACTS devices at necessary nodes. On the other hand, these demands can be internally met with the help of inverters on solar panels [7]. The grid tied solar inverters act as a reactive power source to balance the reactive power of the system.

The optimal operation of a power system is required to precede the optimal planning of facilities like generating plants, reactive power compensation, and transmission networks. In order to handle the large scale optimal power flow problem, the problem is decomposed into real power (*P*) optimization problem and reactive power (*Q*) optimization problem [8]. The *P*-problem is to minimize the production cost under the assumption that system voltages are held constant and the *Q*-problem is to minimize the transmission loss under the assumption that real power generation is held constant. This paper addresses the reactive power dispatch problem for IEEE 30-bus system [8], since the objective is to minimize the transmission line losses.

The network reconfiguration is done to convert a conventional grid into smart grid. Multiple DG units with different *PQ* capacities are integrated to the traditional grid. In [9], different cases are addressed with real and reactive power penetration of the solar plant.

In the integration of solar power, both the siting and sizing of solar Distributed Generators (DGs) have a significant impact on the system losses in a transmission network. This paper presents a novel multiobjective approach for calculating the DG optimum placement and sizing. In [10], optimization for sizing and placement of DGs is done by using PSO algorithm.

Several metaheuristics algorithms were developed for solving the multiobjective reactive power optimization problem. Genetic Algorithm (GA) [11], Evolutionary Programming (EP) [12, 13], Bacterial Foraging Optimization (BFO) [14], Ant Colony Optimization (ACO) [15], Differential Evolution (DE) [16], and recently Gravitational Search Algorithm (GSA) [17] are some of the most common optimization algorithms. The paper [18] deals with the GA for optimum siting and sizing of DGs. Deshmukh et al. [19] have formulated VAR control problem that minimizes the combined reactive power injection by DGs.

Many literatures address the optimal siting and sizing of multiple DGs by any one of the algorithms or combinations of algorithms. Some papers have found the optimal location of DGs by weak (WK) bus placement method or Loss Sensitivity Factor (LSF) method. Some references analyze the impact of increasing the number of DGs. But, this research paper analyzes all the above-said aspects simultaneously.

In this paper, the problem of optimal DG location and sizing is divided into two steps. In the first step, optimal size of DG to be placed at each bus is found out by PSO metaheuristics with the assumption that all 30 buses have solar generation subjected to the inequality constraints of line limits, solar generation real power constraints, and DG size constraints. The results obtained through PSO are checked for reverse power flow by negative load approach. The buses which satisfy the negative loading conditions are the possible locations for DG placement. Again this search of optimal location is fine-tuned by weak (WK) bus placement method and Loss Sensitivity Factor (LSF) method and the results are analyzed. Then, in the second step, optimal sizing of DGs is done by three nature inspired algorithms, namely, Particle Swarm Optimization (PSO), Gravitational Search Algorithm (GSA), and hybrid PSOGSA subjected to many equality and inequality constraints. An augmented multiobjective function with real power loss, reactive power loss, and voltage deviation is used as a fitness function.

Apart from optimal allocation and sizing, this paper analyzes the work in two scenarios. In one aspect the effect of increasing the number of DGs is analyzed. In the second scenario, different *PQ* capacities of DGs supplying real power alone, reactive power alone, and both real and reactive power are also discussed and compared.

Thus the concepts of smart grid, network reconfiguration, and integration of renewable and reactive power optimization with soft computing techniques are all discussed under a single tree. And several aspects are compared and analyzed in this paper.

## 2. Problem Formulation for DG Sizing

The optimal size of DG to be placed at each bus is found out using PSO algorithm. The connection between the DG unit and a bus is modelled as negative *PQ* load in load flow analysis.

First it is assumed that all 30 buses of the system have solar generation. The PSO algorithm is used to find out the size of DG that can be placed at each node. Choosing an objective function as in (1) and considering DG's real power generation as the control variable, the optimal value of DG size is obtained.

**Figure 1:**
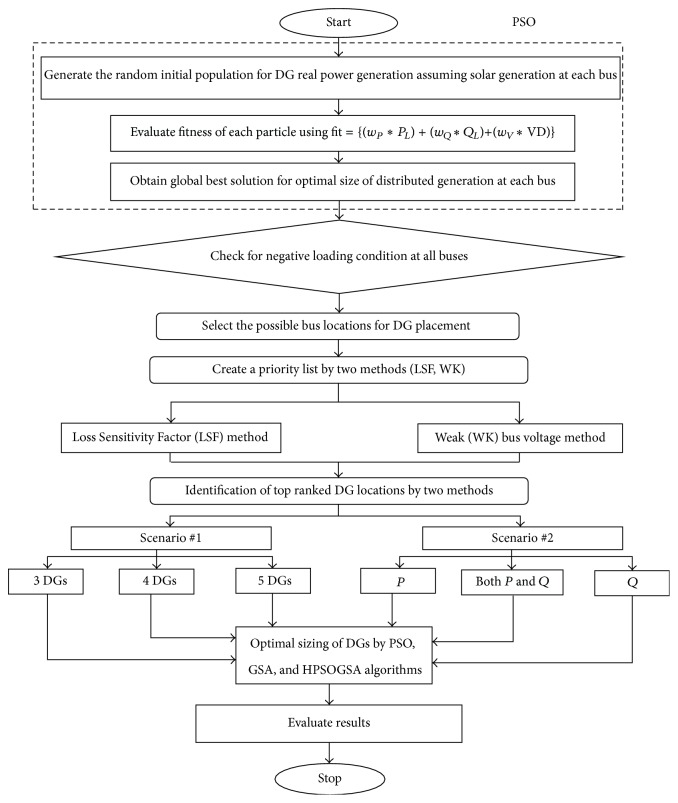
Flowchart showing the proposed method.

As shown in Figure 1, the flowchart explains the overall concept of the paper. The first half inside the dotted box belongs to this part of the section. The PSO algorithm, being more efficient, gives the better results. This nature inspired swarm intelligence algorithm achieves the best size of solar DG to be placed in the system.

The optimal size of DG at each node is determined by PSO. For that, the following multiobjective function, which uses weighted sum of single objective functions, is to be minimized using PSO algorithm.

### 2.1. Objective Function

The objective or fitness function of the ORPD problem tends to minimize the real power losses, reactive power losses, and voltage deviations subjected to the equality and inequality constraints:

(1) $$\begin{array}{c} f=\min\left\{\;\left(\;w_{P}*P_{L}\;\right)+\left(\;w_{Q}*Q_{L}\;\right)+\left(\;w_{V}*VD\;\right)\;\right\}, \end{array}$$

where *P*_*L*_ is the real power loss, *Q*_*L*_ is the reactive power loss, and VD is the voltage deviation and weighing factor for *w*_*P*_ is 0.35, for *w*_*Q*_ is 0.1, and for *w*_*V*_ is 0.55 and sum of all three is maintained as 1. The value of weighing factor is based on the importance of the values in the objective function. Since voltage deviations are of greater concern, it is given higher value and then the real and reactive power losses, respectively. The individual variables, *P*_*L*_, *Q*_*L*_, and VD, are as follows.

*(i) Real Power Loss (P*_*L*_ *).* The total real power losses of the system are given in

(2) $$\begin{array}{c} P_{L}=\overset{N_{l}}{\underset{k=1}{\sum }}G_{k}\left(\;V_{i}^{2}+V_{j}^{2}-2V_{i}V_{j}\cos\left(\;\delta_{i}-\delta_{j}\;\right)\;\right), \end{array}$$

where *N*_*l*_ is the total number of transmission lines in the system; *G*_*k*_ is the conductance of the line *k*; *V*_*i*_ and *V*_*j*_ are the magnitudes of the sending end and receiving end voltages of the line; *δ*_*i*_ and *δ*_*j*_ are angles of the end voltages.

*(ii) Reactive Power Loss (Q*_*L*_ *).* The total reactive power loss of the system is given by

(3) $$\begin{array}{c} Q_{L}=\overset{N_{l}}{\underset{k=1}{\sum }}B_{k}\left(\;V_{i}^{2}+V_{j}^{2}-2V_{i}V_{j}\sin\left(\;\delta_{i}-\delta_{j}\;\right)\;\right), \end{array}$$

where *N*_*l*_ is the total number of transmission lines in the system; *B*_*k*_ is the susceptance of the line *k*; *V*_*i*_ and *V*_*j*_ are the magnitudes of the sending end and receiving end voltages of the line; *δ*_*i*_ and *δ*_*j*_ are angles of the end voltages.

*(iii) Load Bus Voltage Deviation (VD).* Bus voltage magnitude is maintained within the allowable limit to ensure quality service. As shown in (4), voltage profile is improved by minimizing the deviation of the load bus voltage from the reference value (it is taken as 1.0 p.u.):

(4) $$\begin{array}{c} VD=\overset{N_{PQ}}{\underset{k=1}{\sum }}\left|\;\left(\;V_{k}-V_{ref}\;\right)\;\right|, \end{array}$$

where *N*_*PQ*_ is the total number of *PQ* buses in the system.

### 2.2. Constraints

The minimization problem is subjected to the equality and inequality constraints as follows.

*(i) Equality Constraints*

*Load Flow Constraints.* The real and reactive power flow constraints are according to (5) and (6), respectively, as given below:

(5) PGi−PDi−Vi∑j=1nbVjGij cos⁡θij+Bijsin⁡θij=0,

(6) QGi−QDi−Vi∑j=1nbVjGijsin⁡θij+Bijcos⁡θij=0,

where *n*_*b*_ is the number of buses, *P*_*G*_ and *Q*_*G*_ are the real and reactive power generation of generator, *P*_*D*_ and *Q*_*D*_ are the real and reactive load of the generator, *G*_*ij*_ and *B*_*ij*_ are the mutual conductance and susceptance between bus *i* and bus *j*, and *θ*_*ij*_ is the voltage angle difference between bus *i* and bus *j*.

*(ii) Inequality Constraints*

*Line Limits Constraints.* The line thermal flow limits are subjected to

(7) $$\begin{array}{c} S_{l}\leq S_{l}^{max},\;\forall l=\left\{1,2,3,\ldots ,L\right\}, \end{array}$$

where *S*_*l*_ is the thermal limit of each line and *L* is the number of lines in the system.

*Solar Generation Real Power Constraints. *Consider

(8) $$\begin{array}{c} P_{GSn}^{min}\leq P_{GSn}\leq P_{GSn}^{max},\;\forall n,\;\;n=\left\{1,2,3,\ldots ,N\right\}, \end{array}$$

where *P*_GS*n*_ is the real power supplied by DG and *N* is the number of DGs.

*DG Size Constraint. *To obtain a reasonable and economic solution, the size of solar generators added at each node should be so small or so high with respect to total load value. As found in various literatures [20], only 30% of renewable energy penetration is allowable for effective performance of the system. And thus the solar generation at each bus is as follows:

(9) $$\begin{array}{c} \text{1\% }L\leq S\leq \text{5\% }L. \end{array}$$

*L* is the total load value and *S* is the solar capacity.

Steps to find the optimal size of solar DG at each location using PSO are as follows:

(1) Assume that all the 30 buses of the system have solar power generation.
(2) The solar DG supplying real power alone is subjected to the inequality constraints, that is, 1% to 5% of total load at each node. DG unit is modelled as negative *PQ* load in load flow analysis.
(3) The PSO algorithm generates random values for the size of DGs and the algorithm runs with 50 populations and up to 500 iterations.
(4) The algorithm gives out the optimal size of DG to be placed at each node. That also achieves the global optimal fitness values.
(5) These values are taken for further processing of the results.

The solution obtained is carried out to the second part of the work.

## 3. Problem Formulation for Optimal Siting and Sizing

The results obtained from the PSO are checked for negative load approach [10], since DG is added to load terminals. This is to ensure that the power flow studies do not end up with reverse power flow. That is, when each DG is added to the load terminal it supplies the immediate load of that particular bus and returns the remaining power demand to the conventional generators. In that case the following condition should be satisfied by DG at each bus:

(10) $$\begin{array}{c} P_{D_{i}}-P_{{GS}_{i}}>0;\;\forall i=\left\{1,2,3,\ldots ,N\right\}, \end{array}$$

where *P*_*D*_*i*__ is the real power demand at bus *i*, *P*_GS_*i*__ is the real power generated by solar DG at bus *i*, and *N* is the number of solar generators.

When a particular bus satisfies the above condition, then it is suitable for DG placement. If not the corresponding location is not suitable for DG placement. Thus the number of candidate buses opt for DG placement is reduced providing easier solution to find the optimal location.

In [21], Celli et al. have formulated a multiobjective function for optimal sizing and siting with the best compromise between various costs. The effect of ordering DGs location is well addressed in [22] which proves that the order in which the DGs are placed has a significant impact on the system losses.

And the negative load approach determines the buses that are capable of DGs placement and the priority list determines the order of buses to which the DGs are placed among the possible candidate buses.

### 3.1. Finding the Priority Location of DG

The two methods used for creating the priority list are (i) Loss Sensitivity Factor method and (ii) weak bus placement method.

*(i) Loss Sensitivity Factor (LSF) Method.* The Loss Sensitivity Factor method is the best method to find out the order of buses for DG placement [23, 24]. Loss sensitivity can be simply defined as “the ratio of change in total loss of the system when subjected to a small disturbance to the value of disturbance that causes the change.”

Among the selected candidate buses the order of DG allocation is found out by LSF which reduces the search space for allocation problem [25, 26]. The following equation defines the numerical evaluation of LSF:

(11) $$\begin{array}{c} LSF=\frac{\left({loss}_{\text{with DG}}-{loss}_{\text{without DG}}\right)}{\text{size of DG}}. \end{array}$$

NR power flow is run for system with DG and system without DG. Then the priority list is created and buses are placed according to the descending order of the LSF values. The bus with highest sensitivity is first selected for placing DG. The number of DGs to be placed depends on the total allowable penetration to the system [27, 28].

*(ii) Weak (WK) Bus Placement Method.* The weak bus placement is a simple but effective method of ordering buses for DG placement. According to this method NR power flow is performed for base case of the system and the voltage magnitude of each bus is noted down.

Now the priority list is created in ascending order of the bus voltages and the DG is first placed at the weakest bus of the system [29]. But the number of buses in which the DG is to be placed is a separate issue which will be discussed later.

### 3.2. Mathematical Formulation of a Problem for Optimal Sizing

*(1) Objective Function.* The objective function of this problem is to find the optimal settings of reactive power control variables which minimize the real power losses, reactive power losses, and voltage deviation. Hence, the objective function is expressed as in

(12) $$\begin{array}{c} f=\min\left\{\;\left(\;w_{P}*P_{L}\;\right)+\left(\;w_{Q}*Q_{L}\;\right)+\left(\;w_{V}*VD\;\right)\;\right\}, \end{array}$$

where the representation of all the variables is already discussed in Section 2.

*(2) Constraints*

*(i) Equality Constraints*

*Load Flow Constraints.* The real and reactive power constraints are according to (13) and (14), respectively, as given below:

(13) PGi−PDi−Vi∑j=1nbVjGij cos⁡θij+Bijsin⁡θij=0,

(14) QGi−QDi−Vi∑j=1nbVjGijsin⁡θij+Bijcos⁡θij=0,

where the representation of all the variables is already discussed in Section 2.

After addition of renewable energy source, the above equation becomes

(15) PG−PD−Ploss+PSG=0,QG−QD−Qloss+QSG=0,

where *P*_*G*_ and *Q*_*G*_ are the conventional real and reactive power generation, *P*_*D*_ and *Q*_*D*_ are the total real and reactive power demand, *P*_loss_ and *Q*_loss_ are the total system losses, and *P*_SG_ and *Q*_SG_ are the real and reactive power generation of solar DG.

*(ii) Inequality Constraints*

*Generator Bus Voltage (V*_*G*_*i*__ *) Inequality Constraint.* Consider

(16) $$\begin{array}{c} V_{G_{i}}^{min}\leq V_{G_{i}}\leq V_{G_{i}}^{max},\;i\in n_{g}. \end{array}$$

*Load Bus Voltage (V*_*L*_*i*__ *) Inequality Constraint.* Consider

(17) $$\begin{array}{c} V_{L_{i}}^{min}\leq V_{L_{i}}\leq V_{L_{i}}^{max},\;i\in n_{PQ}. \end{array}$$

*Switchable Reactive Power Compensation (Q*_*C*_*i*__ *) Inequality Constraint.* Consider

(18) $$\begin{array}{c} Q_{C_{i}}^{min}\leq Q_{C_{i}}\leq Q_{C_{i}}^{max},\;i\in n_{c}. \end{array}$$

*Reactive Power Generation (Q*_*G*_*i*__ *) Inequality Constraint.* Consider

(19) $$\begin{array}{c} Q_{G_{i}}^{min}\leq Q_{G_{i}}\leq Q_{G_{i}}^{max},\;i\in n_{g}. \end{array}$$

*Transformer Tap Setting (T*_*i*_ *) Inequality Constraint.* Consider

(20) $$\begin{array}{c} T_{i}^{min}\leq T_{i}\leq T_{i}^{max},\;i\in n_{t}, \end{array}$$

where *n*_*g*_, *n*_*PQ*_, *n*_*c*_, and *n*_*t*_ are the numbers of generator buses, load buses, switchable reactive power sources, and tap settings.

*Solar Generators Real Power Constraints.* Consider

(21) $$\begin{array}{c} P_{GSn}^{min}\leq P_{GSn}\leq P_{GSn}^{max},\;\forall n,\;\;n=\left\{1,2,3,\ldots ,N\right\}, \end{array}$$

where *P*_GS*n*_ is the real power supplied by DG and *N* is the number of DGs.

*Solar Generators Reactive Power Constraints.* Consider

(22) $$\begin{array}{c} Q_{GSn}^{min}\leq Q_{GSn}\leq Q_{GSn}^{max},\;\forall n,\;\;n=\left\{1,2,3,\ldots ,N\right\}, \end{array}$$

where *Q*_GS*n*_ is the reactive power supplied by DG and *N* is the number of DGs.

## 4. Proposed Work

Integrating the renewable source to grid or the conversion of the traditional grid into smart grid involves several essential steps. The placement of renewable sources, their size, and amount of penetration into the system are all to be considered. The smart grid possesses five most significant characteristics like being adaptive, predictive, integrated, interactive, optimized, and secured [30]. In this paper the grid achieves all the five perspectives by integrating solar energy, placement of solar energy, and performing reactive power optimization to maintain voltage profile and thus having secured and reliable power system.

Solar DG is found to be the best DG that adapts the grid among several renewable sources. And also this research includes the grid interactive solar inverters and their behavior when operated in a grid [31]. The grid tied solar inverters act as a reactive power source to balance the reactive power of the system [7]. In the [32], optimal siting and sizing of DGs are found by combined GA/PSO algorithms.

The proposed work consists of two scenarios:

 Scenario #1: number of DG placements.
 Scenario #2: DG supplying various *PQ* capacities.

### 4.1. Scenario #1: Number of DG Placements

This scenario discusses the number of DGs to be placed on the system and effect of increased number of DGs on the system [18].

The number of DGs to be placed depends on the load demand of the system and maximum allowable size of DG. The maximum allowable size of DG is up to 25 to 30% of the total load as found in various literatures [18, 20]. In this paper, the maximum numbers of DGs are taken as 5. So it is proposed to select 5 numbers of buses among 30-bus system. Then for each bus it is advisable to take maximum of 5% load in order to satisfy the total load of 25% amongst 5 buses. Also minimum numbers of DGs are chosen as 3. In this aspect, this paper does not include constant solar DG penetration of 25%. That means total 25% of load is not divided amongst 3 buses. Instead in each bus solar DG size of 1% to 5% load is maintained independent of number of DGs. So this work attains the fact that minimum of 15% load is satisfied by including 3 numbers of DGs and maximum of 25% load is met by including 5 numbers of DGs.

Therefore the number of DGs is selected as 3, 4, and 5, respectively. And they are placed on the top priority ordered by LSF and weak bus method. Now three nature inspired metaheuristics algorithms PSO [33], GSA [34, 35], and hybrid PSOGSA [36, 37] are used to optimize the solar sizing. In [38], economic dispatch problem in a microgrid is addressed with cost minimization.

*(i) Basic Concepts of PSO [33]. *PSO has been developed through simulation of simplified social models. The features of the method are as follows:

- Based on swarms like fish schooling and a flock of birds.
- Simple and less time consuming.
- Solving nonlinear optimization problems with continuous variables.

The convergence is provided by the acceleration term in (23).

The modified velocity of each agent can be calculated using the current velocity and the distances from *pbest* and *gbest* are as shown below:

(23) vik+1=wvik+c1rand⁡pbesti−sik+c2rand⁡gbest−sik,

where *v*_*i*_^*k*^ is the velocity of agent *i* at *k*th iteration, *v*_*i*_^*k*+1^ is the modified velocity of agent, rand represent functions that generate independent random numbers which are uniformly distributed between 0 and 1, *s*_*i*_^*k*^ is the current position of agent at *k*th iteration, *pbest*_*i*_ is the *pbest* of agent *i*, *gbest* is the *gbest* of the group, *w* is the inertia weight factor used to control the impact of the previous history of velocities *v*_*i*_^*k*^ on the current velocity *v*_*i*_^*k*+1^, and *c*_1_ and *c*_2_ are the acceleration factors named cognitive and social parameters determine the influence of *pbest*_*i*_ and *gbest* in determining the new solutions.

And the updated position can be calculated from the following equation:

(24) $$\begin{array}{c} s_{i}^{k+1}=s_{i}^{k}+v_{i}^{k+1},\;i=\left\{1,2,3,\ldots ,N_{tm}\right\}, \end{array}$$

where *N*_*tm*_ is the number of swarms and *k* represents the iteration.

*(ii) Basic Concepts of GSA [34, 35]. *GSA is a novel heuristic optimization method which has been proposed by Rashedi et al. in 2009 [35]. The basic physical theory from which GSA is inspired is from Newton's theory. The GSA could be considered as an isolated system of masses. It is like a small artificial world of masses obeying the Newtonian laws of gravitation and motion.

The convergence is reached indirectly by the acceleration term in (25). The acceleration of any mass is equal to the force acted on the system divided by mass of inertia.

The velocity and position of the agents for next (*t* + 1) iteration are calculated using the following equations:

(25) vidt+1=randi⁡vidt+aidt,

(26) xidt+1=xidt+vidt+1,

where *v*_*i*_^*d*^(*t* + 1) is the updated velocity of the particle at *i*th position, *v*_*i*_^*d*^(*t*) is the previous velocity, *a*_*i*_^*d*^(*t*) is the acceleration of the particle at *i*, and *x*_*i*_^*d*^ is the position of the particle.

*(iii) Basic Concepts of Hybrid PSOGSA [36, 37].* Two algorithms can be hybridized in high level or low level with relay or coevolutionary method as homogeneous or heterogeneous. In this paper, low level coevolutionary heterogeneous hybrid method is used. The hybrid is low level because of the combination of the functionality of both algorithms. It is coevolutionary because this algorithm does not use both algorithms one after another. In other words, they run in parallel. It is heterogeneous because there are two different algorithms that are involved to produce final results. The main idea is to integrate the ability of exploitation in PSO with the ability of exploration in GSA to synthesize both algorithms' strength.

The main objective is to combine the social thinking ability of PSO (*gbest*) with the local search capability of GSA, hence achieving a new formula for hybrid PSOGSA for velocity updating as

(27) vit+1=wvit+c1′rand⁡ acit+c2′rand⁡gbest−xit,

where *v*_*i*_(*t*) is the velocity of agent *i* at iteration *t*, *c*_*j*_′ is a weighting factor, *w* is a weighting function, rand is a random number between 0 and 1, ac_*i*_(*t*) is the acceleration of agent *i* at iteration *t*, and *gbest* is the best solution so far. Position update is done by the following formula:

(28) $$\begin{array}{c} x_{i}\left(\;t+1\;\right)=x_{i}\left(\;t\;\right)+v_{i}\left(\;t+1\;\right). \end{array}$$

### 4.2. Scenario #2: DG Supplying Various *PQ* Capacities

This paper deals with solar power and grid tied solar inverters in which both real power and reactive power of solar energy are considered. And this scenario deals with 4 types of cases [9, 18, 39].

The four different cases as shown below are separately analyzed with respect to LSF and WK method in order to find out the location of solar power and they are also analyzed with respect to number of DGs using different optimization algorithms to find out the sizing of solar power and the results are discussed in the next section:

(i) Type  1: system without DG (initial case).
(ii) Type  2: DG supplying real power alone (*P*).
(iii) Type  3: DG supplying reactive power alone (*Q*).
(iv) Type  4: DG supplying both real and reactive power.

All the four types are tested with above-mentioned three algorithms and results are evaluated.

## 5. Results and Discussions

**Figure 2:**
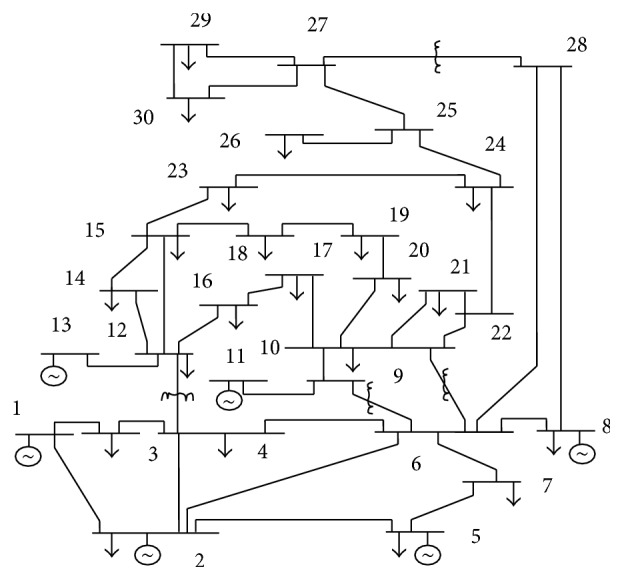
Single line diagram of IEEE 30-bus test system.

In this section of the paper, the entire work is explained in a precise manner. The standard IEEE 30-bus system [8] as in Figure 2 [37] is used as a test system and the results are evaluated. The test system consists of 30 buses of which 6 generating buses are present including slack bus and remaining 24 are load buses. 41 lines, 4 tap changing transformers, and 9 shunt capacitors are present in this test case. MATPOWER open source power system software is used to run NR power flow. The initial real power loss of the system is 5.8316 MW and initial voltage deviation is 0.9819.

The proposed work is divided into two steps. In the first step, optimal size of DG at each node is found by PSO algorithm assuming that all the 30 buses have solar generation. And then results obtained through PSO are checked for reverse power flow by negative load approach to find the possible bus locations for DG placement. Then, the search for optimal location of DGs is fine-tuned by two methods, namely, weak (WK) voltage bus placement and Loss Sensitivity Factor (LSF) method. In order to emphasize point on the effect of increasing the number of solar generators, this paper analyses and compares the result with numbers 3, 4, and 5 of DGs which are placed using priority location found from WK and LSF methods. Further the *PQ* capacities of solar DGs are also analyzed with DG supplying *P* alone (real power alone), *Q* alone (reactive power alone), and both *P* and *Q* (both real and reactive power). Thus several aspects like size of DGs, number of DGs, location of DGs, order of DG placement, and *PQ* capacities of DGs are all discussed under one roof in this paper.

### 5.1. Optimal Size of DG Using PSO

The PSO algorithm is used to find out the size of DG to be placed on the system. At each bus solar DG penetration is allowed and size of solar power is taken as a control variable. The size is limited between 2.834 MW and 11.336 MW which is 1% to 5% of total load.

Table 1 illustrates the optimal size of the solar DG at each bus to be included satisfying the multiobjective function of minimizing the real power losses, reactive power losses, and voltage deviations.

Then the size of the DG is tested for negative load approach. That is, NR power flow is run after placing DG at each bus. In the literature listed in [40] Kansal et al.'s work is limited to reverse power flow and they have not included negative load approach. But this paper provides best results and does not have reverse power flow.

The buses that withstand negative load effect are 2, 4, 5, 7, 8, 12, 15, 19, 21, 24, and 30. Among these buses the best location and the best combination for DG placement are selected as in Tables 1 and 2.

### 5.2. Priority List Creation

The priority list is created to order the DG placement (shown in Table 2). It is done by two methods (1) Loss Sensitivity Factor (LSF) method and (2) weak (WK) bus placement method:

1. The LSF method of placement is used because of the sensitivity towards loss. Since this paper aims for loss minimization with voltage enhancement, LSF method of finding DG location is most appropriate. The buses are placed in decreasing order of loss sensitivity; that is, the highly sensitive bus is first given with DG siting. Thus the order of buses is obtained as in Table 2.
2. The weak (WK) bus voltage method is the simplest and easy method for bus placement. The buses with weak voltage profile are provided with the DG. This is in order to improve the voltage profile of the system and to obtain minimum voltage deviations. The ordering is done as in Table 2.

The bus number in which solar DG can be fit is highlighted, that is, the DGs which sustain negative load approach. Therefore those buses are provided with DG in the order of LSF or WK method. Now the LSF method brings out the priority order as 30, 24, 19, 21, 15, 12, 7, 5, 4, 8, and 2, whereas by WK method the priority order is 30, 24, 21, 19, 15, 12, 7, 5, 8, 4, and 2.

### 5.3. Location and Sizing

After finding out the order and location of buses the number of DGs and type of DG are to be found. The cases may be 3 DGs, 4 DGs, and 5 DGs based on number of placements. Here the size of DG is same and is subjected to the same limits. And type of DGs concept is provided in this paper to answer the following question: “What purpose is DG placement meant for?” According to this concept four cases are dealt with “no DG, *P* alone, *Q* alone, and both *P* and *Q* combined.” All these cases are tested with both LSF and WK bus ordering.

#### 5.3.1. Control Variables and Its Ranges

Consider the following:

(1) Real power supplied by DG *P*_SG_, 2.834 to 11.336 MW.
(2) Reactive power supplied by DG *Q*_SG_, 1.262 to 6.31 MVAR.
(3) Voltage magnitude of PV buses, 0.9 to 1.1 p.u.
(4) Tap settings, 0.9 to 1.1 p.u.
(5) Shunt capacitors, 0 to 10 MVAR.

Allowed total solar power generation is from 5% to 25% of the total load of the system. For an IEEE 30-bus system, the total real power demand is 283.4 MW and reactive power is 126.2 MW. Therefore each DG is subjected to 1% to 5% of total demand and maximum of 5 DGs are considered to satisfy 5% to 25% of total demand.

**Figure 3:**
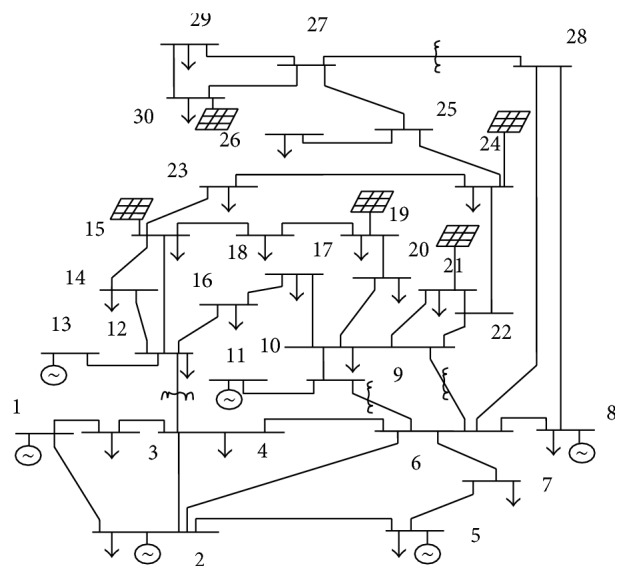
Test system after addition of DGs at 5 locations.

Four types of system cases are evaluated:

1. No DG: In this case there is no renewable penetration and NR load flow is run with the basic case with conventional generators. Here the values of fitness function are evaluated for the base case using GSA, PSO, and hybrid PSOGSA algorithms. And the results are tabulated in Table 4.
2. Three DGs placed: In this case totally three DGs are placed in the system. By LSF method, DG location is prioritized at bus numbers 30, 24, and 19. By WK method, it is found to be at 30, 24, and 21. This brings out reduced loss results than no DG case.
3. Four DGs placed: In this case 4 DGs are placed at location of bus numbers 30, 24, 19, and 21 under LSF and 30, 24, 21, and 19 under WK. This case exhibits more reduced losses than previous case.
4. Five DGs placed (as shown in Figure 3): Under LSF, DG location is found to be at bus numbers 30, 24, 19, 21, and 15 and under WK it is at 30, 24, 21, 19, and 15. Five-DG case brings out the most wondering results as in Table 3.

It is to be noted that there is only slight variation in the order of placement by both methods, but the results obtained have shown ridiculous performance. The DG supplying various *PQ* capacities concept is discussed next:

(1) *P* alone: This system consists of multiple DGs supplying real power alone located under LSF and WK bus methods. The system with multiple DGs supplying *P* alone is optimized using 3 algorithms and the results are tabulated in Table 4.
(2) *Q* alone: This scheme consists of multiple DGs supplying reactive power alone located under LSF and WK bus method is solved under all three algorithms. That is, the DG is placed as a reactive power compensating device. This is a worst case with high power loss incurred compared to other cases. Also it is not economical to include DG just for providing reactive power alone. So this case will be the worst case among all and the results are tabulated in the Appendix.
(3) Both *P* and *Q*: Under this scheme, multiple DGs are placed satisfying LSF and WK methods meant for providing both real and reactive power. This case is analyzed with 3, 4, and 5 DGs using 3 algorithms. This provides a trustworthy way of DG installation. Here the real and reactive power needs of the system are compensated at the same time. The inverters of the solar DG act as reactive power sources. This condition suits the main objective of the paper and it aims to compensate both the powers of the system. Even though it has slightly higher fitness values than *P* alone case this condition is best, considering its effectiveness, and will provide a new approach to renewable energy integration.

In order to reduce the length of paper, all the results are tabulated in Appendix except the best case (5-DG LSF). For 5-DG LSF case the results are tabulated in Table 3 using 3 algorithms. It is found that the 5-DG placement gives the most optimal loss under LSF order of placement with hybrid PSOGSA algorithm with DG supplying *P* alone. Anyway DGs supplying both *P* and *Q* type are also equally effective.

### 5.4. Selection of Best Algorithm with Increased Solar Power Generation

**Figure 4:**
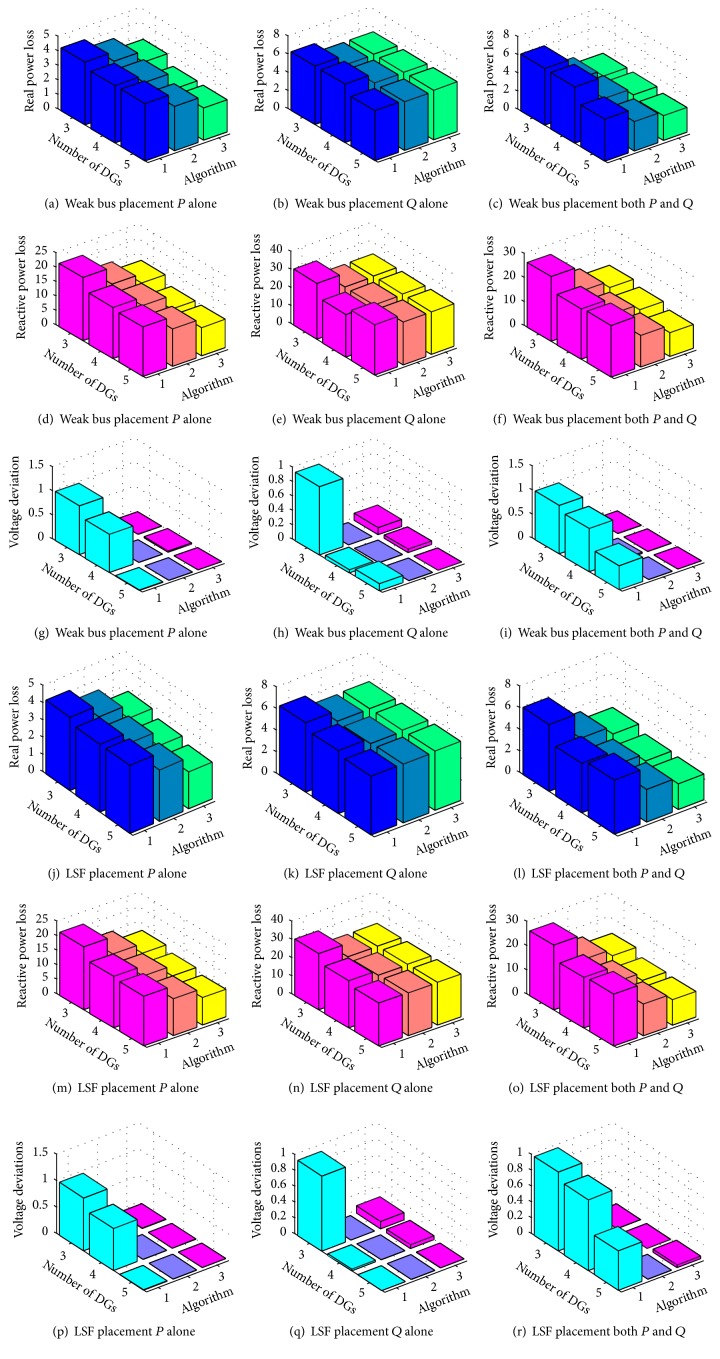
Algorithms used versus number of DGs placed for *P*_*l*_, *Q*_*l*_, and VD.

To explain the results in an easy and quick way 3D plots are drawn (Figures 4(a)–4(r)). The graphs shown in Figures 4(a)–4(r) describe the values of real power loss (*P*_*l*_), reactive power loss (*Q*_*l*_), and voltage deviations (VD) for 3, 4, and 5 DGs' placements under all 3 algorithms. The results bring out a conclusion that 5 DGs' case is best compared to 3 and 4 DGs' placements. And this gives us a hope that increased level of solar penetration increases the system stability thereby reducing losses. And this case is further improved when it is solved by hybrid PSOGSA algorithm.

### 5.5. Selection of Best Approach with DG Type

**Figure 5:**
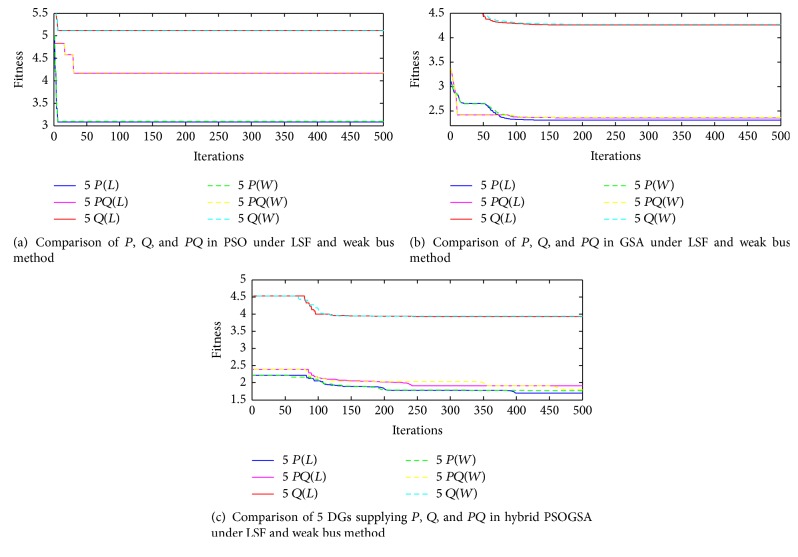
Fitness values of *P* alone, *Q* alone, and both *P* and *Q* cases under LSF and WK bus methods.

So far it is found that 5-DG placement under hybrid is the best result. The best DG placement approach and best DG type are discussed in this section. Therefore the fitness function value of 5-DG placement under *P* alone, *Q* alone, and both *P* and *Q* is plotted with LSF and WK bus method. The results are illustrated in Figures 5(a)–5(c).

From the graphs obtained, it is obvious that the *P* alone case is best under LSF placement method. That is, if a solar DG is placed in the system with constant power factor and is supplying real power alone this yields best results.

But this would not be a wanted result because the aim is to make the solar DG integration for reactive power demand. Therefore DG supplying both *P* and *Q* type yields better results than the initial case. Therefore the best solar DG placement is with *P* alone and better placement is with both *P* and *Q* and the worst placement is with *Q* alone. But one interesting point to note is that the LSF (green, blue, and yellow lines in Figure 5) turns out to be the best ordering in all three algorithms. Since the fitness function is to minimize the total loss, the LSF method gives the best order compared to WK bus method.

**Figure 6:**
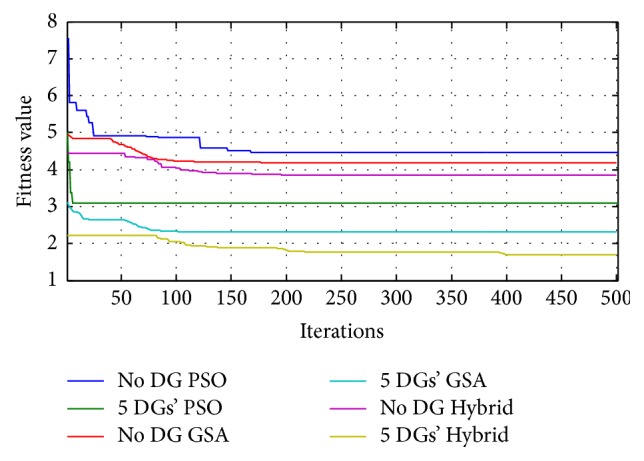
Comparison of no DG case with 5 DGs under LSF in *P* alone.

The entire research consists of huge number of data calculations and various comparisons to prove the originality and efficiency of the work done. To reduce length, only important and best results among the achieved results are tabulated and highlighted. The comparison of no DG with 5-DGs case under three nature inspired algorithms is shown in Figure 6. All the no DG cases have high fitness value and all 5-DGs cases have minimum fitness value. Also the results show that hybrid PSOGSA possesses a better capability to escape from local optimums with faster convergence than the standard PSO and GSA. Table 4 shows the comparison of no DG case with 5 DGs placed case under LSF ordering for solar DG supplying *P* alone and DG supplying both *P* and *Q* type. Also the values of all the control variables are listed in Table 4.

## 6. Conclusion

The research carried out in this paper is worthwhile and includes several important points to be noted. Basically optimal siting and sizing of solar DG in IEEE 30-bus system are the main concept but there are several parallel researches which are carried out to enrich the results.

Initially PSO algorithm is used to find out the optimal size of DG and the size of DG is tested for negative load impact (to avoid reverse power flow). Now the candidate bus for placing DGs is narrowed. Further the order of DG placement is done under LSF and WK bus method of ordering. Then the work with number of DGs' placement is elaborated with 3, 4, and 5 DGs' placements. And DG supplying various *PQ* capacities is also discussed with *P* alone, *Q* alone, and both *P* and *Q* cases. All the results are evaluated using 3 different algorithms (PSO, GSA, and hybrid PSOGSA). Below are the final conclusions that are visibly found from the research:

(1) PSO gives the best result for optimal DG sizing in a very short span of time.
(2) Negative load approach check prevents reverse power flow condition throughout the process.
(3) LSF ordering serves best compared to WK bus method, ensuring that the total losses are minimized.
(4) 5 DGs' placement case is the best, proving that increased level of DG penetration decreases the total power losses in the system and maintains flatter voltage profile.
(5) DG supplying real power (*P*) alone is the best suitable case for the system denoting that when DG is under unity power factor environment, it gives best results.
(6) DGs supplying both real (*P*) and reactive (*Q*) power also give better results and pave way for a new technology of “grid interactive solar power for real and reactive power source.” Here there is no need for maintaining the power factor at unity and any change in voltage or reactive power of the system can be gently met with multiple DGs which will provide more stability to the system.
(7) Algorithm-wise the hybrid PSOGSA performs in a super way by combining the advantages of both PSO and GSA.

Thus the best optimal values of fitness function, real power losses, reactive power losses, voltage deviations, and control variables are obtained from hybrid PSOGSA algorithm solving 5 DGs' case placed in LSF order with DGs supplying real power (*P*) alone.

Thus the conventional system is reconfigured by optimally integrating the solar DG at globally best locations with globally best size. This makes the grid work smarter which comes under smart grid environment. Future works involve further improvisation that allows increased solar penetration by different ways to achieve better results.

## Figures and Tables

**Table 1:** Size of DGs from PSO.

Bus number	Optimal size (MW)
1	4.984768
2	4.358467
3	5.21738
4	6.469943
5	5.496305
6	8.266242
7	10.21549
8	7.167197
9	7.500256
10	6.786414
11	3.778254
12	7.562593
13	10.56481
14	7.046026
15	6.007336
16	7.425125
17	10.94804
18	6.917231
19	4.613798
20	7.559447
21	10.33308
22	5.125532
23	3.969423
24	6.293101
25	6.45544
26	4.166992
27	5.630553
28	7.452936
29	5.480901
30	7.336586

**Table 2:** Sequence of priority list by WK and LSF method.

Bus number	LSF	Priority list	Bus voltage (*V*)	Priority list
1	6.05*e* − 14	30	1.050	30
**2**	1.07**e** − 01	**29**	**1.040**	**27**
3	2.35*e* − 01	27	1.028	26
**4**	2.44**e** − 01	**26**	**1.022**	**25**
**5**	2.47**e** − 01	**25**	**1.010**	**21**
6	2.63*e* − 01	24	1.017	24
**7**	2.83**e** − 01	**23**	**1.006**	**20**
**8**	2.30**e** − 01	**28**	**1.010**	**22**
9	4.49*e* − 01	19	0.976	17
10	5.02*e* − 01	16	0.955	14
11	4.29*e* − 01	20	1.050	29
**12**	4.76**e** − 01	**18**	**0.998**	**19**
13	4.12*e* − 01	21	1.050	28
14	5.61*e* − 01	13	0.997	18
**15**	5.75**e** − 01	**12**	**0.968**	**15**
16	5.19*e* − 01	17	0.972	16
17	5.26*e* − 01	15	0.954	13
18	5.95*e* − 01	7	0.950	12
**19**	5.88**e** − 01	**8**	**0.943**	**9**
20	5.76*e* − 01	11	0.945	10
**21**	5.76**e** − 01	**10**	**0.941**	**7**
22	5.77*e* − 01	9	0.941	8
23	6.23*e* − 01	4	0.947	11
**24**	6.14**e** − 01	**5**	**0.927**	**6**
25	5.90*e* − 01	6	0.920	4
26	6.64*e* − 01	3	0.901	2
27	5.56*e* − 01	14	0.926	5
28	3.04*e* − 01	22	1.012	23
29	6.95*e* − 01	2	0.903	3
**30**	7.38**e** − 01	**1**	**0.891**	**1**

**Table 3:** Best outcome among all cases.

5 DGs' LSF									

Algorithm	PSO			GSA			Hybrid		

DG type	*P*	*Q*	*PQ*	*P*	*Q*	*PQ*	*P*	*Q*	*PQ*

FIT	3.0851	4.260241	4.165504	2.309231	4.260241	2.358722	**1.696819**	4.295037	1.920378
*P*_*L*_ (MW)	3.930302	5.447339	5.004235	2.973936	5.447339	3.022499	**2.149479**	5.496311	2.407849
*Q*_*L*_ (MVAR)	17.06019	23.53672	21.41433	12.68353	23.53672	13.00848	**9.444453**	23.70543	10.57728
VD	0.006318	5.11*E* − 10	0.495616	1.63*E* − 10	5.11*E* − 10	6.15*E* − 11	1.02**E** − 04	0.001427	0.036186
*P* solar (MW)	35.01449	—	34.57517	35.23621	—	35.72001	**46.64924**	—	41.69984
% *P* solar	12.35515	—	12.20013	12.43338	—	12.6041	**16.46056**	—	14.71413
*Q* solar (MVAR)	—	15.41866	13.55552	—	15.41866	15.43187	—	15.40234	14.53383
% *Q* solar	—	12.21764	10.7413	—	12.21764	12.22811	—	12.2047	11.51651

**Table 4:** Optimal values obtained for best result cases.

Control variables	No DG			5 DGs' LSF					
	PSO	GSA	Hybrid	PSO		GSA		Hybrid	
				*P*	*PQ*	*P*	*PQ*	*P*	*PQ*
*V*_*G*_1__	1.089917	1.043908	1.099979	1.069308	0.953278	1.027006	1.018297	1.08685	1.074454
*V*_*G*_2__	1.082366	1.034023	1.091815	1.088762	0.943393	1.021787	1.012454	1.082779	1.068675
*V*_*G*_5__	1.062927	1.00884	1.069204	1.1	0.969738	1.0009	0.991337	1.062865	1.047696
*V*_*G*_8__	1.039571	1.011822	1.068482	1.058558	0.994771	1.01*E* + 00	9.94*E* − 01	1.067363	1.053259
*V*_*G*_11__	1.050594	1.022672	0.985823	1.070703	0.974405	1.024211	1.011202	0.9792	0.970688
*V*_*G*_13__	1.01784	1.032264	0.988852	0.946661	1.03617	1.023102	1.00859	0.980954	0.979823
*T*_6–9_	1.007764	0.992142	1.081123	1.065734	0.96849	0.985923	0.990601	1.094339	1.085628
*T*_6–10_	1.013321	0.991287	1.079838	1.016578	1.006752	0.982872	0.999366	1.087027	1.07876
*T*_4–12_	0.974232	0.988311	1.090329	1.096735	1.010581	0.988205	0.983372	1.09214	1.075721
*T*_27-28_	0.998855	0.996503	1.018112	1.1	1.005154	1.003624	0.99675	1.005436	1.006862
*Q*_10_	0	5.085415	6.710543	1.013802	1.02632	4.587534	5.458556	6.135134	5.5659
*Q*_12_	5.910549	4.815389	6.514192	5.623678	5.629074	4.953474	4.783481	6.015178	5.631306
*Q*_15_	9.409826	5.035168	6.211642	9.501841	9.18421	5.153297	5.083737	6.233104	5.558516
*Q*_17_	5.640918	5.263779	6.238567	5.019682	5.075339	5.216868	5.443653	6.13873	5.372718
*Q*_20_	3.559025	5.482562	6.625873	3.710988	3.733716	5.562014	5.448493	5.979799	5.498727
*Q*_21_	5.846265	5.747952	6.653561	5.959905	5.733319	5.359788	5.347674	6.197945	5.600752
*Q*_23_	10	6.245089	6.680471	9.704982	9.699361	5.659589	5.957551	6.072773	5.471516
*Q*_24_	5.489716	4.813535	6.524242	5.12668	5.156616	4.408785	4.87739	6.059574	5.515494
*Q*_29_	3.073788	6.131744	6.515898	2.492969	2.435573	6.338891	6.457947	6.089195	5.529888
*PG*_*S*_30__	—	—	—	7.845027	7.710418	6.914579	7.578882	8.035948	7.544586
*PG*_*S*_24__	—	—	—	10.5418	10.54352	7.564736	7.497634	8.003554	7.523266
*PG*_*S*_19__	—	—	—	6.197235	5.876226	6.588308	6.687485	7.937735	11.336
*PG*_*S*_21__	—	—	—	4.147673	4.009441	6.719616	6.538963	11.336	7.597863
*PG*_*S*_15__	—	—	—	6.430637	6.435573	7.44897	7.417048	11.336	7.69812
*QG*_*S*_30__	—	—	—	—	3.052677	—	3.311614	—	3.348694
*QG*_*S*_24__	—	—	—	—	2.054185	—	2.841042	—	3.261832
*QG*_*S*_19__	—	—	—	—	2.623982	—	2.817567	—	3.313153
*QG*_*S*_15__	—	—	—	—	2.464426	—	3.53734	—	1.262468
*QG*_*S*_21__	—	—	—	—	3.360252	—	2.924316	—	3.347687

The units of *V*_*G*_, *T*, *Q*, *PG*, and *QG* in the table are in p.u., p.u., MW, and MVAR.

**Table 5:** 

Control variables	5 DGs' WK bus								
	PSO			GSA			Hybrid		
	*P*	*Q*	*PQ*	*P*	*Q*	*PQ*	*P*	*Q*	*PQ*
*V*_*G*_1__	1.069308	1.069308	0.953278	1.019529	1.031666	1.018056	1.085504	5	1.085358
*V*_*G*_2__	1.088762	1.088762	0.943393	1.014656	1.02243	1.012203	1.081343	1.02243	1.080559
*V*_*G*_5__	1.1	1.1	0.969738	0.994113	0.999328	0.991059	1.061518	0.999328	1.060591
*V*_*G*_8__	1.06*E* + 00	1.058558	9.95*E* − 01	9.98*E* − 01	1.00*E* + 00	9.94*E* − 01	1.065719	1.000208	1.062606
*V*_*G*_11__	1.070703	1.070703	0.974405	1.029595	1.013144	1.011856	0.978619	1.013144	0.965969
*V*_*G*_13__	0.946661	0.946661	1.03617	1.030549	1.023832	1.006507	0.98228	1.023832	0.967785
*T*_6–9_	1.065734	1.065734	0.96849	0.975017	0.990016	0.98121	1.088784	0.990016	1.1
*T*_6–10_	1.016578	1.016578	1.006752	0.992393	0.993312	0.997504	1.089788	0.993312	1.1
*T*_4–12_	1.096735	1.096735	1.010581	0.977333	0.984654	0.986355	1.087525	0.984654	1.1
*T*_27-28_	1.1	1.1	1.005154	0.996589	1.001132	0.999475	1.004579	1.001132	1.010224
*Q*_10_	1.013802	1.013802	1.02632	4.580024	4.724464	5.449149	6.102514	4.724464	5.706719
*Q*_12_	5.623678	5.623678	5.629074	4.959731	5.004031	4.782598	5.936364	5.004031	5.469174
*Q*_15_	9.501841	9.501841	9.18421	5.172064	5.169386	5.073348	6.327842	5.169386	5.417166
*Q*_17_	5.019682	5.019682	5.075339	5.216286	5.26975	5.476113	6.193466	5.26975	5.591361
*Q*_20_	3.710988	3.710988	3.733716	5.562951	5.543824	5.421283	6.209261	5.543824	5.624744
*Q*_21_	5.959905	5.959905	5.733319	5.347173	5.346397	5.328151	5.975524	5.346397	5.727526
*Q*_23_	9.704982	9.704982	9.699361	5.653493	5.625959	5.959699	6.149491	5.625959	5.375455
*Q*_24_	5.12668	5.12668	5.156616	4.394021	4.349062	4.898907	6.190531	4.349062	5.538482
*Q*_29_	2.492969	2.492969	2.435573	6.346391	6.290148	6.442072	6.072827	6.290148	5.602689
*PG*_*S*_30__	7.845027		7.710418	6.901596		7.568974	8.192465		7.556758
*PG*_*S*_24__	10.5418		10.54352	7.545859		7.472385	7.997332		11.336
*PG*_*S*_19__	6.197235		5.876226	6.574641		6.69055	8.783369		7.577594
*PG*_*S*_21__	4.147673		4.009441	6.728393		6.555974	8.022007		11.336
*PG*_*S*_15__	6.430637		6.435573	7.464848		7.424602	11.3355		7.574928
*QG*_*S*_30__		3.551065	3.052677		3.027613	3.315499		3.027613	3.253077
*QG*_*S*_24__		4.691007	2.054185		3.297964	2.830617		3.297964	3.344049
*QG*_*S*_19__		2.969467	2.623982		2.889797	2.813561		2.889797	3.387994
*QG*_*S*_21__		1.945957	3.360252		2.942343	2.936526		2.942343	3.319405
*QG*_*S*_15__		2.860872	2.464426		3.25268	3.540268		3.25268	3.326865

The units of *V*_*G*_, *T*, *Q*, *PG*, and *QG* in the table are in p.u., p.u., MW, and MVAR.

**Table 6:** 

Algorithm	PSO			GSA			Hybrid		

DG type	*P*	*Q*	*PQ*	*P*	*Q*	*PQ*	*P*	*Q*	*PQ*

5 DGs' WK bus									
FIT	3.096217	5.114391	4.177373	2.36139	4.2768	2.364621	1.764437	4.2768	1.800886
*P*_*L*_ (MW)	3.948507	6.48354	5.023242	3.047111	5.474296	3.029391	2.234555	5.474296	2.291437
*Q*_*L*_ (MVAR)	17.0867	27.91454	21.45659	12.94902	23.60796	13.04334	9.823366	23.60796	9.988834
VD	0.010127	0.097633	0.497416	3.79*E* − 07	9.16*E* − 07	2.20*E* − 09	1.02*E* − 05	9.16*E* − 07	2.12*E* − 07
*P* solar (MW)	35.16237		34.57517	35.21534		35.71248	44.33067		45.38128
% *P* solar	12.35515		12.20013	12.42602		12.60144	15.64244		16.01315
*Q* solar (MVAR)		16.01837	13.55552		15.4104	15.43647		15.4104	16.63139
% *Q* solar		12.69284	10.7413		12.21109	12.23175		12.21109	13.1786

3 DGs' WK bus									
FIT	4.264306	5.883197	5.437968	2.894293	4.23752	2.993451	2.319683	4.335688	2.489634
*P*_*L*_ (MW)	4.330255	6.442337	6.252752	3.687836	5.411755	3.820455	2.918493	5.469429	3.154463
*Q*_*L*_ (MVAR)	21.90277	31.06185	26.93665	16.0355	23.43406	16.56292	12.98041	23.68064	13.85381
VD	1.02*E* + 00	0.949443	1.010617	3.15*E* − 11	2.16*E* − 10	6.45*E* − 11	3.07*E* − 04	0.096953	3.48*E* − 04
*P* solar (MW)	24.11545		24.10796	21.3087		21.26735	28.35612		27.09767
% *P* solar	8.509333		8.506692	7.518949		7.504356	10.00569		9.561632
*Q* solar (MVAR)		10.67152	7.689319		9.323842	9.659002		0.096953	10.82097
% *Q* solar		8.456037	6.092963		7.388148	7.653726		7.297119	8.574461

3 DGs' LSF									
FIT	4.212065	5.883197	5.397336	2.81944	4.251188	2.989552	2.212357	3.921414	2.450767
*P*_*L*_ (MW)	4.251328	6.442337	6.206426	3.569999	5.437177	3.802894	2.776769	5.484566	3.046115
*Q*_*L*_ (MVAR)	21.7197	31.06185	26.78419	15.6994	23.48176	16.58536	12.38338	23.71063	13.84625
VD	1.003874	0.949443	0.99394	1.12*E* − 09	3.89*E* − 08	6.37*E* − 06	0.003908	0.091727	2.97*E* − 06
*P* solar (MW)	24.11545		24.10796	21.341		21.29026	31.00476		23.9273
% *P* solar	8.509333		7.689319	7.530345		7.512442	10.94028		8.442944
*Q* solar (MVAR)		10.67152	8.506692		9.313316	9.662229		9.208964	5.900359
% *Q* solar		8.456037	6.092963		7.379807	7.656283		7.297119	4.675403

4 DGs' WK bus									
FIT	3.590047	4.310123	4.204349	2.692499	4.204649	2.574001	1.820807	4.310123	2.163678
*P*_*L*_ (MW)	3.943014	5.477871	4.608111	3.458567	5.360118	3.222565	2.284012	5.477871	2.728538
*Q*_*L*_ (MVAR)	17.72519	23.6799	20.97923	14.81996	23.28607	14.34279	10.08463	23.6799	12.08686
VD	0.795406	0.045233	0.897432	8.34*E* − 06	2.46*E* − 06	2.15*E* − 02	0.023527	0.045233	6.88*E* − 06
*P* solar (MW)	28.27499		28.13829	28.2066		31.82575	42.07584		34.21096
% *P* solar	9.962176		9.928825	9.952928		11.22998	14.8468		12.07162
*Q* solar (MVAR)		12.15449	10.65566		12.38437	12.42506		12.15449	13.52161
% *Q* solar		9.631134	8.443475		9.813289	9.84553		9.631134	10.71443

4 DGs' LSF									
FIT	3.576975	4.651419	4.188804	2.579767	4.209986	2.567546	1.925261	4.309961	2.001535
*P*_*L*_ (MW)	3.924537	5.831084	4.584043	3.298612	5.36723	3.227293	2.429493	5.477474	2.53162
*Q*_*L*_ (MVAR)	17.67696	25.99136	20.92145	14.25248	23.31448	14.33873	10.74905	23.67911	11.15468
VD	0.792166	0.020734	0.89499	8.04*E* − 06	1.43*E* − 05	7.49*E* − 03	6.01*E* − 05	0.045335	9.06*E* − 07
*P* solar (MW)	28.27499		28.13829	28.19092		31.84273	38.83614		38.21796
% *P* solar	9.977059		9.928825	9.947396		11.23597	13.70365		13.48552
*Q* solar (MVAR)		13.01001	10.65567		12.38567	12.39378		12.15449	12.44819
% *Q* solar		10.30904	8.443475		9.814316	9.820745		9.631134	9.863862

## Appendix

See Tables 5 and 6.
